# Convergent evolution of *SWS2* opsin facilitates adaptive radiation of threespine stickleback into different light environments

**DOI:** 10.1371/journal.pbio.2001627

**Published:** 2017-04-11

**Authors:** David A. Marques, John S. Taylor, Felicity C. Jones, Federica Di Palma, David M. Kingsley, Thomas E. Reimchen

**Affiliations:** 1 Department of Biology, University of Victoria, Victoria, British Columbia, Canada; 2 Stanford University School of Medicine, Department of Developmental Biology, Stanford, California, United States of America; 3 Friedrich Miescher Laboratory of the Max Planck Society, Tübingen, Germany; 4 Earlham Institute and University of East Anglia, Department of Biological Sciences, Norwich, United Kingdom; The Institute of Science and Technology Austria, Austria

## Abstract

Repeated adaptation to a new environment often leads to convergent phenotypic changes whose underlying genetic mechanisms are rarely known. Here, we study adaptation of color vision in threespine stickleback during the repeated postglacial colonization of clearwater and blackwater lakes in the Haida Gwaii archipelago. We use whole genomes from 16 clearwater and 12 blackwater populations, and a selection experiment, in which stickleback were transplanted from a blackwater lake into an uninhabited clearwater pond and resampled after 19 y to test for selection on cone opsin genes. Patterns of haplotype homozygosity, genetic diversity, site frequency spectra, and allele-frequency change support a selective sweep centered on the adjacent blue- and red-light sensitive opsins *SWS2* and *LWS*. The haplotype under selection carries seven amino acid changes in *SWS2*, including two changes known to cause a red-shift in light absorption, and is favored in blackwater lakes but disfavored in the clearwater habitat of the transplant population. Remarkably, the same red-shifting amino acid changes occurred after the duplication of *SWS2* 198 million years ago, in the ancestor of most spiny-rayed fish. Two distantly related fish species, bluefin killifish and black bream, express these old paralogs divergently in black- and clearwater habitats, while sticklebacks lost one paralog. Our study thus shows that convergent adaptation to the same environment can involve the same genetic changes on very different evolutionary time scales by reevolving lost mutations and reusing them repeatedly from standing genetic variation.

## Author summary

When organisms colonize a new environment in replicate, natural selection often leads to similar phenotypic adaptations. Such “convergent evolution” is known from both distant relatives, e.g., sea cows and whales adapting to an aquatic life, and from multiple populations within a species, but the causing genetic changes are rarely known. Here, we studied how a fish, the threespine stickleback, repeatedly adapted its color vision to living in red light–dominated blackwater lakes. Using multiple natural populations and a 19-y evolution experiment, we found selection on a blue light–sensitive visual pigment gene. One allele of this gene with a red-shifted light sensitivity facilitated repeated blackwater colonization. Surprisingly, two amino acid changes responsible for the red-shift have independently occurred 198 million years earlier, after the gene was duplicated in the ancestor of all spiny-rayed fish and modified into blue- and red-shifted gene copies. While other fish species today use these two gene copies to adapt to clear- and blackwater, stickleback have lost a copy and reevolved these mutations on different alleles of the same gene causing convergent adaptation to these habitats. Thus, we conclude that the same genetic changes can be responsible for convergent evolution on very different time scales.

## Introduction

Successful colonization of a new habitat requires adaptation to a multitude of different selection pressures. When similar habitats are colonized in replicate by different populations or species, phenotypic adaptation is often convergent [1], and this is most striking in adaptive radiations in multiple lakes or on several islands [2]. Whether a similar phenotypic adaptation is caused by selection on variants present in a shared ancestor due to recurrent mutation or due to changes in different genes, however, is still poorly understood [3, 4]. Only recently, genetic and population genomic studies have started to unravel the evolutionary mechanisms of phenotypic convergence [5–12].

An adaptive radiation with replicate habitat colonization is found among threespine stickleback (*Gasterosteus aculeatus*) inhabiting the Haida Gwaii archipelago, British Columbia, Canada. Since the retreat of the ice sheets 12,000 years ago, and likely before that [13–15], marine stickleback have colonized hundreds of freshwater habitats independently in different watersheds and adapted in predictable ways to highly divergent “ecological theatres” [16]. One major predictor of natural selection in the Haida Gwaii stickleback radiation is the spectrum of visible light [16]. Most Haida Gwaii lakes are either oligotrophic clearwater lakes featuring full-spectrum light to blue-shifted light with increasing depth, or they are dystrophic blackwater lakes, stained by dissolved tannins leading to a red-shifted light spectrum [16–18]. Blackwater lakes are extreme, almost “nocturnal” visual environments, as both downwelling short-wavelength light and almost all up- or sidewelling light is absorbed, leaving only downwelling red light in a small cone above the focal animal.

Evolutionary adaptation to blackwater lakes in Haida Gwaii stickleback had consequences for multiple traits: stickleback have evolved larger body sizes and reduced lateral plates, both maximizing burst velocity and agility to escape from a predator on short reaction distance, and the former increasing postcapture resistance to predators [16]. Not only natural selection by predators, but also sexual selection interacts with light spectra: blackwater stickleback males have replaced red with black nuptial throat color, which maximizes contrast to the blue eye and against the background via reversed counter-shading [17]. And both traits, the black throat and blue eyes, are preferred by females choosing their mates [19]. Also, color vision was adapted to the blackwater light spectrum: double cones in the retina of stickleback from blackwater systems express only red light–sensitive photopigments instead of one red and one green light–sensitive photopigment, increasing the stickleback’s visual sensitivity to dominant red light [18]. Expression differences are heritable and replicated between independently colonized blackwater lakes [18], but the genetic mechanisms underlying these differences are still unknown.

Here, we study the evolutionary history of color vision genes during adaptation to blackwater environments. To perceive color, vertebrates use a combination of membrane-bound photosensitive proteins, called visual opsins, that are expressed in cone cells in the retina (i.e., cone opsins) and have peak sensitivities at different wavelengths [20]. Vertebrates have evolved large opsin repertoires via gene duplication and divergence [21, 22]. Previous research showed that opsins have evolved in response to the visual environment by sequence or expression divergence (i.e., “spectral tuning,” [18, 23–30]). Adaptation of color vision has been found both between [31–34] and within species [35–37], sometimes without gene duplication [38] or without sequence divergence [39]. Remarkably, spectral tuning of opsins has led to convergent adaptation by recurrent mutations, leading to the same amino acid sequences in distantly related species, families, orders, or phyla [31, 40–43]. These observations together with extensive biochemical and mutagenesis study of opsin proteins have led to the identification of several “key site” substitutions [44–46], from which genotypes the light absorption phenotype can be predicted. Both functional experiments and the evolutionary history of opsins thus show that there are many different, functionally tractable “molecular roads” to color vision adaptation.

Most ray-finned fish possess large repertoires of eight or more cone opsin genes, originating from a combination of ancient and recent lineage-specific gene duplication events, facilitating adaptation to a diversity of visual environments in aquatic systems [21, 22]. However, threespine stickleback have only four cone opsins: the UV sensitive *SWS1*, a single blue-sensitive *SWS2*, a single green-sensitive *RH2*, and a red-sensitive *LWS* [18, 22]. This is an impoverished repertoire compared to most other fish species, and when compared to relatives among spiny-rayed fish, two *SWS2* paralogs and one *RH2* paralog have been lost [22, 47].

Although we know of parallel and heritable expression differences between blackwater and clearwater habitats [18] and between marine and freshwater habitats [36], the targets of selection in the genome are unknown and it is unclear whether spectral tuning of amino acids is involved in adaptation to divergent freshwater habitats [6, 36]. Here, we assess whether and which cone opsin genes have experienced recent selection using two types of evidence. First, we use whole genomes from one oceanic and 27 freshwater fish from across the Haida Gwaii adaptive radiation and from coastal British Columbia, including 15 clearwater and 12 blackwater populations derived from 18 watersheds independently colonized by marine ancestors over the last 12,000 y [13]. Second, we use whole genomes from a selection experiment in which 100 adult stickleback from a blackwater lake were transferred to a barren clearwater pond, from which 11 individuals were resampled after 19 y [48]. Then, we screen cone opsin genes for amino acid variation with predictable effects on color vision and test whether selection on such coding changes or noncoding variation has facilitated the colonization of blackwater habitats. Finally, we compare the molecular mechanisms of adaptation to blackwater habitat among Haida Gwaii threespine stickleback to other blackwater-inhabiting fish species and therein uncover convergent evolution on vastly different time scales.

## Results

### Signature of selection around linked blue- and red-sensitive opsins

**Fig 1:**
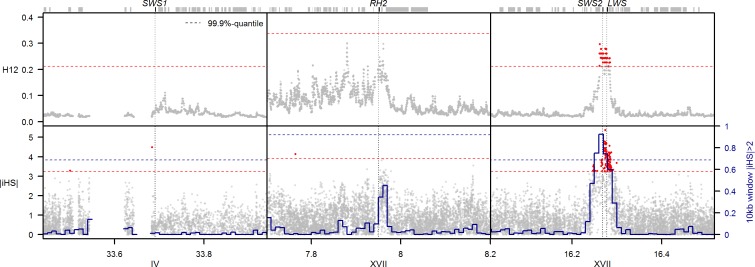
Scan for selective sweeps around cone opsins in Haida Gwaii threespine stickleback. One of the strongest outlier regions for H12 and iHS in the genome of Haida Gwaii stickleback from 28 populations is centered on *SWS2* and *LWS*, indicating a past selective sweep in this region. No such signature is found around the other two cone opsins. Grey dots show H12 (upper panel) and absolute iHS (lower panel) values for each SNP with a minor allele frequency (MAF) greater 5%, with top 0.1% outlier SNPs highlighted as red dots. The proportion of iHS-SNPs with a value greater than 2 per 10 kb window is shown as a blue line. The 99.9%-quantile boundaries are shown as red- and blue-dashed lines for SNPs and windows, respectively. Genomic coordinates in Mb based on an improved version of the stickleback reference genome [51] are given on the x-axis. The position of the four cone opsin genes is highlighted with black boxes and vertical dashed lines; grey boxes indicate other genes. Depicted values for H12, iHS, window-iHS, and 99.9%-quantile boundaries can be found in S1 Data.

**Fig 2:**
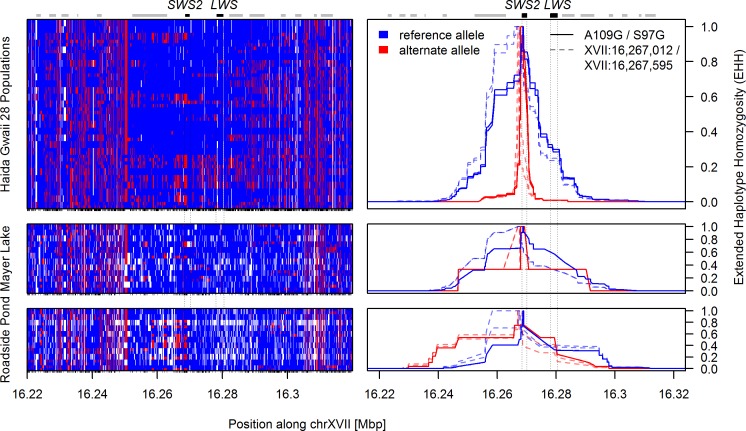
Haplotype structure and extended haplotype homozygosity around *SWS2* and *LWS*. Left panels show phased haplotypes, right panels the decay of haplotype homozygosity around selected SNPs, each for the adaptive radiation (upper panels) and the selection experiment dataset (middle and lower panels). The selective sweep signature seen in Fig 1 is caused by a long run of reference alleles (blue, upper left panel). This led to an extended haplotype homozygosity (EHH) signal at SNPs in the top H12-window (dashed lines in top right panel): haplotype homozygosity decays slowly around the reference allele (blue) for these SNPs, but rapidly around their alternate alleles (red). Decay is similar for *SWS2* key amino acid substitutions A109G and S97G (full lines). In the selection experiment (middle/lower panels), the same EHH decay is found for the reference haplotype, but the alternate haplotype shows reduced decay, in particular in the transplant population Roadside Pond. Rows in the left panel show haplotypes with imputed SNPs and monomorphic sites with missing data (white). Columns represent SNPs with color code relative to the threespine stickleback reference genome, a freshwater stickleback female [6]. Gene positions are indicated with boxes above the figure. The haplotype matrix and EHH values can be found in S1 Data.

We sequenced the genomes of 58 threespine stickleback from 25 freshwater populations on Haida Gwaii, two freshwater sites from coastal British Columbia, and one marine site (Table 1), resulting in a dataset of 7,888,602 high-quality SNPs with transition to transversion ratio (Ts/Tv) 1.26 (see materials and methods). We split this dataset into an “adaptive radiation” partition, containing single individuals from each natural population on Haida Gwaii, two coastal British Columbia populations, and one mid-Pacific marine population (*n* = 28 individuals, 6,526,842 SNPs, Ts/Tv = 1.31), and a “selection experiment” partition, containing 12 individuals from the blackwater Mayer Lake source population and 11 individuals from the clearwater Roadside Pond transplant population (*n* = 23 individuals, 4,180,622 SNPs, Ts/Tv = 1.26). We scanned the adaptive radiation dataset for evidence of selective sweeps at the four cone opsin genes, using the haplotype-based statistics iHS and H12 [49, 50] and their variation across the genome to identify outlier regions. We identified a prominent outlier region for both iHS and H12 metrics in Haida Gwaii sticklebacks, suggesting a selective sweep centered on a genomic region containing both the blue- and red-sensitive opsin genes, *SWS2* and *LWS* (Fig 1). In contrast, H12 and iHS around the green- and UV-sensitive opsins *RH2* and *SWS1* were not significantly different from the genome-wide expectation, although both statistics are elevated around *RH2*, which is in the vicinity of a more heterogeneous genomic background due to reduced recombination in this region. The selective sweep signature centered on *SWS2* and *LWS* is caused by haplotypes with a long run of reference alleles, uninterrupted by recombination and thus leading to an extended haplotype homozygosity (EHH) across the 28 populations for the reference “sweep haplotype” (Fig 2). The same EHH signature for the sweep haplotype was found in the selection experiment within the blackwater population Mayer Lake. However, 13 generations after the transfer of Mayer Lake fish into the clearwater habitat of Roadside Pond, the alternate haplotype shows a stronger EHH signature (Fig 2), indicative of the alternate haplotype quickly rising to high frequency in the selection experiment.

**Table 1:** Population information.

Abbreviation	Population	Habitat	Depth	Area	T400	Blackwater	Watershed	Year	Number
ANSR	Anser	lake	4.0	18.0	76.0	no	Kumara	2009	1
BK70	Banks 70	lake	3.0	5.0	91.0	no	Banks 70	2010	1
BKW2	Banks W2	lake	2.0	2.0	85.0	no	Banks W2	2010	1
BOUL	Boulton	lake	4.0	15.0	78.1	no	Masset inlet	2010	2
BRNT	Branta	lake	4.0	3.0	70.0	yes	Sangan	2009	1
COAT	Coates	lake	30.0	90.0	94.5	no	Coates	2009	1
DARW	Darwin	lake	15.0	13.9	90.0	no	Darwin	2009	1
DAWS	Dawson	lake	4.0	1.0	82.0	no	Dawson	2009	1
DRIZ	Drizzle	lake	16.0	97.0	67.0	yes	Sangan	2009	4
EDEN	Eden	lake	50.0	513.0	87.0	no	Naden	2010	1
ESCP	Escarpment	lake	50.0	97.0	93.6	no	Barry	1993	1
GOLD	Gold Creek	stream	–	–	50.2	yes	Mayer	2009	2
LAUR	Laurel	lake	2.0	2.0	70.0	yes	Sangan	1993	1
LUTE	Lutea	lake	2.0	3.0	93.9	no	Burnaby	2003	1
MAYR	Mayer Lake	lake	20.0	489.9	57.1	yes	Mayer	2004	12
MNYN	Menyanthes	lake	5.0	6.0	82.0	no	Hughes	2009	1
MDPC	Mid-Pacific	marine	–	–	–	no	–	1993	1
POQU	Poque	lake	25.0	17.0	90.8	no	Poque	2009	1
RDSP	Roadside Pond	lake	1.0	0.3	80.0	no	–	2012	11
ROUG	Rouge	lake	2.0	1.2	68.1	yes	Kliki	2000	1
SDPY	Serendipity	lake	2.0	3.0	70.5	yes	Hiellen	2006	1
SKID	Skidegate	lake	4.0	8.5	60.0	yes	Copper	2009	1
SKON	Skonun	lake	20.0	734.0	94.4	no	Sangan	2009	1
SLTC	Solstice	lake	15.0	51.0	68.0	yes	Sangan	2009	1
SLVR	Silver	lake	5.0	11.7	72.2	yes	Kumara	2009	1
SPNC	Spence	lake	30.0	95.0	75.0	no	Hiellen	2009	4
STIU	Stiu	lake	30.0	24.0	92.8	no	Stiu	2009	1
WATT	Watt	lake	2.0	4.0	60.0	yes	Mayer	2009	1
WDPL	Woodpile	lake	2.0	4.0	60.8	yes	Mayer	2009	1

Sample sizes (number) indicate sequenced individuals, lake area is given in hectares, lake depth in meters, and light transmission at 400 nm in percent (T400).

**Fig 3:**
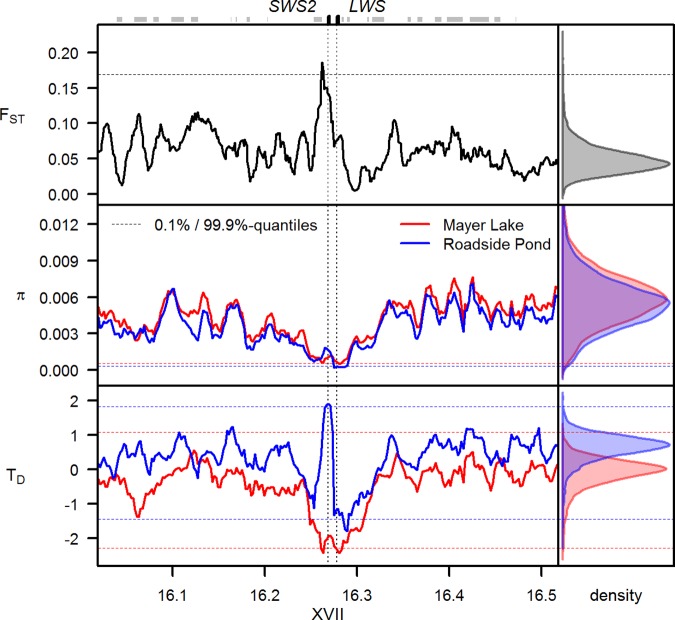
Signature of selective sweep in blackwater-adapted stickleback and “reversed sweep” after transplant to clearwater habitat. Significantly reduced nucleotide diversity (middle left) and Tajima’s D (T_D_, bottom left) in the blackwater source population Mayer Lake compared to genome-wide expectations (right) indicate a selective sweep in this population, consistent with the signature seen across the adaptive radiation (cf. Figs 1 and 2). After transplant to a clearwater habitat, however, the alternate haplotype has increased in frequency (Fig 2), leading to one of the strongest differentiation signals in the genome (F_ST_, top left panel) and a significantly positive Tajima’s D. Such a pattern is consistent with a transient phase of a reversed selective sweep. Dashed horizontal lines indicate the 0.1%- and 99.9%-quantiles for each statistic based on their genome-wide distribution in regions with similar recombination rates (right panels); boxes above the figure indicate the position of genes, with black boxes and vertical dashed lines highlighting the position of the two cone opsins *SWS2* and *LWS*. Sliding-window F_ST_, nucleotide diversity and Tajima’s D values and genome-wide distributions of each statistic can be found in S1 Data.

**Fig 4:**
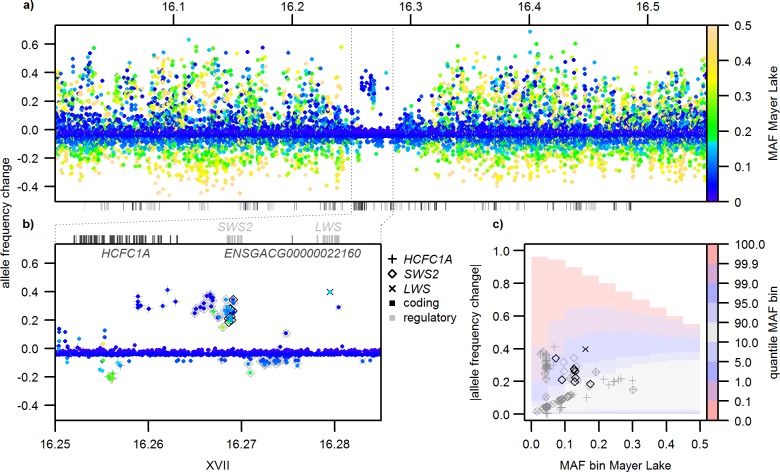
Allele-frequency change around *SWS2* and *LWS* in the selection experiment. Distribution of single SNP allele-frequency change in the selection experiment (Roadside Pond minus Mayer Lake) around the selective sweep region on chromosome XVII (a,b). Note the unexpectedly strong increase of low frequency alleles linked to blue-shifting *SWS2* coding variation after the transplant to a clear water pond. Color codes in (a) and (b) show “starting” allele frequencies, i.e., minor allele frequencies (MAFs) in Mayer Lake, color codes in (c) indicates the genome-wide distribution of allele-frequency change for Mayer Lake MAF bins of 0.05 width. Symbol shapes and colors in (b) and (c) indicate associations with different genes and predicted effects. Black and grey boxes below (a) and above (b) are exons of genes surrounding the sweep region. Depicted allele frequencies and allele-frequency change quantiles can be found in S1 Data.

Patterns of nucleotide diversity, differentiation, site frequency spectra, and allele-frequency change across the genome from the selection experiment data support the presence of a selective sweep signature in the region containing the two opsins *SWS2* and *LWS* and two tightly linked genes, *HCFC1A* and *ENSGACG00000022160* (Figs 3 and 4, S1 Fig). In the blackwater source population Mayer Lake, nucleotide diversity is significantly reduced compared to the genome-wide expectation, and Tajima’s D is strongly negative, as expected under a selective sweep (Fig 3). The transplant into a clearwater habitat, however, has led to an increase in frequency of the alternate haplotype (Fig 2) and therefore to significant differentiation in this region between the two populations, as measured by F_ST_, ranking this region among the highest 0.1% differentiated regions in the genome (Fig 3). While nucleotide diversity and Tajima’s D on *LWS* are still reduced in the source population Mayer Lake from the initial selective sweep, the rising frequency of the alternate haplotype has led to a positive Tajima’s D centered on *SWS2*. These high Tajima’s D values are among the top 0.1% outliers even against the positively shifted genome-wide distribution of Tajima’s D, which was caused by the bottleneck experienced during the population transplant [48]. This positive Tajima’s D is a consequence of both reduced diversity due to the first sweep shared with Mayer Lake (cf. Fig 3) and a rapid increase of the alternate haplotype in the transplant population to a similar frequency as the haplotype favored in Mayer Lake (Fig 2). A transient phase of a “reverse selective sweep” for the alternate haplotype, associated with the shift in light regime in the selection experiment, or the combined effect of a past selective sweep and a bottleneck may have caused this pattern. The fact that this genomic region is among the top 0.1% F_ST_ outlier windows and that linked low-frequency alleles associated with *SWS2* and *LWS* are among the top 1% allele-frequency changes in the genome (Fig 4) support selection for the alternate haplotype in the transplant population. Note that linkage disequilibrium is not increased beyond the region containing the two opsins *SWS2* and *LWS* and the two linked genes, perhaps due to high recombination in this chromosomal segment, making it unlikely that selection on genes further up- or downstream was involved (S1 Fig., [51, 52]).

### Selection on coding variation favoring red-shifted *SWS2*

**Fig 5:**
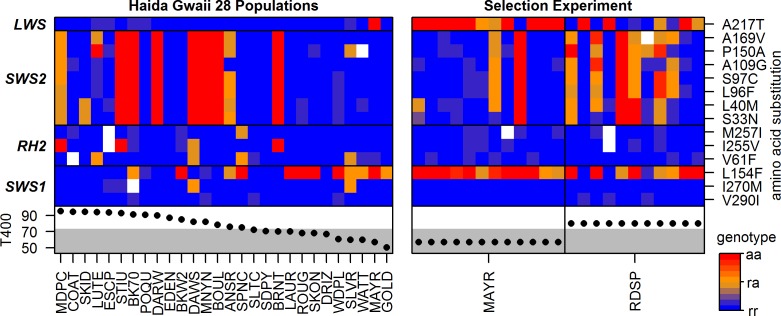
Cone opsin amino acid polymorphisms across the Haida Gwaii stickleback radiation and selection experiment. Note the high frequency and nearly perfect linkage of *SWS2* amino acid polymorphisms. Columns represent single individuals per population, rows represent amino acid polymorphisms. Color codes show the genotype probabilities and amino acid alleles relative to the threespine stickleback reference genome (S97C: reference = S, alternate = C; rr: homozygous for reference amino acid, aa: homozygous for alternate amino acid, ra: heterozygous genotype). Amino acid positions are relative to the bovine rhodopsin protein. Light transmission ratios at 400 nm (T400) for the different water bodies are given in percent in the lower panels, with the grey area representing blackwater lakes [16]. Genotype probabilities can be found in S1 Data, T400 values in Table 1.

**Fig 6:**
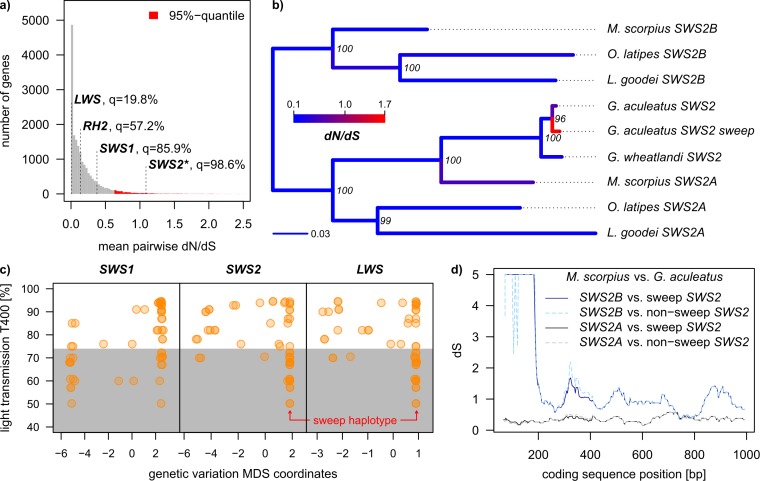
Accelerated protein evolution, evolutionary origin of threespine stickleback *SWS2*, and association with blackwater habitation. (a) *SWS2* shows an unexpected high frequency of amino acid polymorphisms given the total number of mutations in *SWS2*. The distribution of mean pairwise relative protein sequence divergence (mean ratio of non-synonymous to synonymous substitutions [dN/dS]) is based on 17,846 functional protein coding genes in the stickleback genome. Each value is based on pairwise comparisons between all 56 haplotypes in the “adaptive radiation dataset” containing one individual per population from the Haida Gwaii adaptive radiation, two mainland freshwater, and a marine site (see materials and methods). (b) Rooted phylogenetic tree of *SWS2* opsins in stickleback and related taxa, which have retained two ancient *SWS2* paralogs. Both red-shifted (“sweep”) and blue-shifted threespine stickleback haplotypes are derived from the ancestral *SWS2A* paralog, and the haplotype under selection has rapidly accumulated amino acid changes (dN/dS = 1.64). Branches are color coded with maximum-likelihood dN/dS estimates, and node labels show Bayesian branch credibility. (c) Genetic variation at *SWS2*, *LWS*, and *SWS1*, each summarized in single multidimensional scaling (MDS) coordinates, is associated with light transmission at 400nm (T400), but only variation at the sweep haplotype carrying coding and noncoding *SWS2* and noncoding *LWS* variation is associated with the colonization of blackwater habitats. The grey area indicates blackwater lakes, following [16]. (d) Gene conversion did not contribute to molecular convergence between stickleback haplotypes and *SWS2* paralogs in other fish: synonymous divergence (dS) between the two threespine stickleback haplotypes is larger to the *SWS2B* paralog than to the *SWS2A* paralog, except for a region around bp 700–800, a region without intraspecific amino acid variation. Mean pairwise dN/dS values for all genes shown in (a), MDS values for the three opsins *SWS1*, *SWS2*, and *LWS* in (c), and dS values in (d) can be found in S1 Data.

We identified segregating amino acid polymorphisms in all four cone opsins and genes linked to the selective sweep around *SWS2* and *LWS* in both the adaptive radiation and selection experiment datasets (Fig 5). The blue-sensitive *SWS2* opsin contains the highest number of amino acid polymorphisms, with seven alternative amino acid residues occurring at high frequency and in nearly perfect linkage (Fig 5), in contrast to only four synonymous substitutions. The sweep haplotype identified in the adaptive radiation dataset carries the same alleles as the reference genome for all seven amino acid polymorphism in *SWS2*, leading to a nearly perfect association of the *SWS2* polymorphisms with the sweep haplotype (χ^2^ tests, all *SWS2* Bonferroni-corrected *p* < 0.05, Fig 2), while no association is found between the *LWS* polymorphism and the sweep haplotype. Segregating amino acid polymorphisms in *SWS2* are both numerous and occur at high frequency in the adaptive radiation SNP dataset, leading to a high mean pairwise ratio of non-synonymous to synonymous substitutions (dN/dS) estimate of 1.08 between the 56 haplotypes in this dataset, an exceptional value when compared to other functional protein-coding genes in the threespine stickleback genome (Fig 6A).

Thanks to earlier mutagenesis and protein structure studies, we can predict functional consequences for four *SWS2*, one *LWS*, and two *RH2* amino acid polymorphisms. Three *SWS2* polymorphisms are at opsin key sites 96, 97, and 109 and cause shifts in the peak light absorption of *SWS2* [53] and the rod opsin *RH1* [54]. Notably, the sweep haplotype reference alleles at *SWS2*-specific key sites, S97 and A109, lead to a “red-shift” in the absorption spectrum, i.e., a peak absorbance at a longer wavelength, while the alternate haplotype alleles at sites C97 and G109, lead to a “blue-shift,” a shorter wavelength absorption maximum at *SWS2* [53]. Furthermore, these two key sites and three other polymorphic amino acids in *SWS2* (site 40) and *RH2* (sites 179 and 203) face the “retinal binding pocket” of the opsin proteins, in which a functional effect is likely [55]. In *SWS1* and *LWS*, no polymorphism falls into a key or retinal binding pocket site, but the single *LWS* substitution (A217T) replaces a hydrophobic with a hydrophilic residue and may thus have functional consequences. While this polymorphism is not associated with the sweep haplotype in the adaptive radiation dataset (Fig 5), it has increased in frequency linked with the blue-shifted *SWS2* haplotype in the selection experiment alongside noncoding SNPs around *HCFC1A* (Figs 4 and 5).

**Fig 7:**
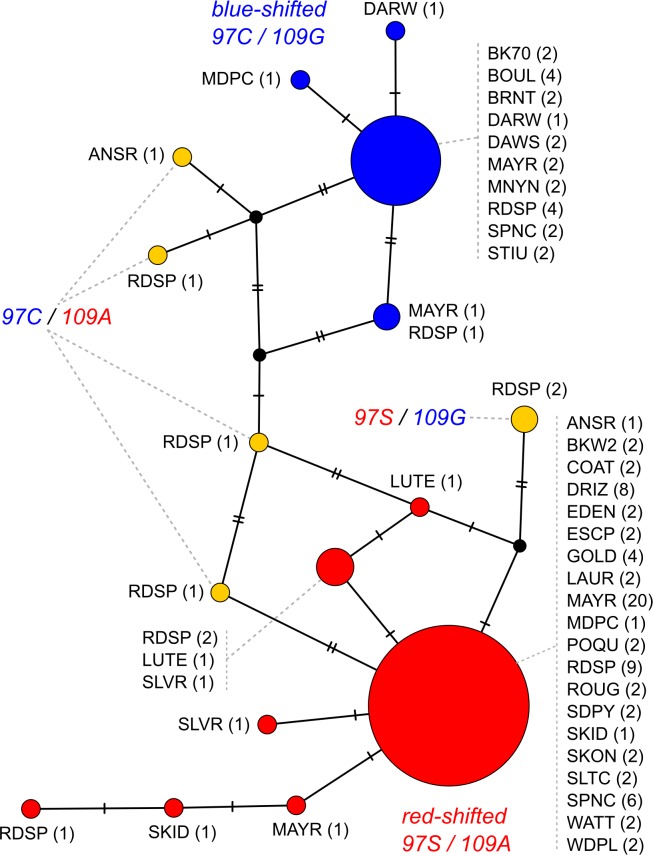
Median-joining network of *SWS2* coding sequence haplotypes. Virtually all populations across the Haida Gwaii adaptive radiation recruited the same red- or blue-shifted *SWS2* allele. Population codes are shown in Table 1, numbers in brackets indicate the number of haplotypes among all 58 sequenced individuals, and the color code indicates haplotypes predicted to be red- or blue-shifted or intermediate, based on the two *SWS2* opsin key sites at position 97 and 109.

Population genomic patterns and functional predictions suggest that the amino acid polymorphisms in *SWS2* are the most likely target of the selective sweep among Haida Gwaii stickleback and again in the selection experiment, while noncoding variation in both opsins and the two linked genes, the latter lacking coding variation, may have hitchhiked on the selected haplotype. Notably, the same haplotype with the same red-shifted *SWS2* key sites is found across the Haida Gwaii adaptive radiation: nearly all populations show identical *SWS2* coding sequence haplotypes, either the same blue-shifted or red-shifted haplotype (Fig 7). Selection thus favored the same red-shifted *SWS2* cone opsin sequence across multiple Haida Gwaii populations and the alternate and widespread blue-shifted *SWS2* haplotype in the selection experiment (Figs 2, 5 and 7).

### Blackwater colonization associated with coding *SWS2* and noncoding *SWS2/LWS* variation

We asked whether the selective sweep for a red-shifted *SWS2* cone opsin was associated with the colonization of blackwater. For this, we tested for an association between genetic variation at the four cone opsins and three predictors and covariates of visual environment: light transmission at 400 nm (T400), lake area, and lake depth. Light transmission at 400 nm is a predictor of light intensity and spectrum, with lower transmission indicating a red-shifted light spectrum in Haida Gwaii lakes [18]. Lake area is a strong predictor of between lake differences in predation regime and might capture the interaction of visual environment and predation landscape influencing selection on color vision [16]. The third covariate, lake depth, is a predictor for variation in light spectra found within a single lake, with more divergent light spectra and thus potential for disruptive selection on color vision in deeper lakes [16, 18]. Genetic variation at cone opsins *SWS2*, *SWS1*, and *LWS* was significantly associated with T400 but not with lake depth or area (Table 2, Fig 6C). When we tested more specifically for an association with blackwater (T400 ≤ 74%), we found only genetic variation at *SWS2* and *LWS*—sweep haplotype variation—to be significantly associated with blackwater (Table 2, Fig 6C). Correlation with blackwater was strongest for four coding and seven noncoding SNPs in *SWS2*, one noncoding SNP also being an upstream regulatory SNP for *LWS* (single SNP χ^2^, *p* < 0.01). In addition, 28 noncoding SNPs around the UV-sensitive *SWS1* opsin showed such a strong correlation, likely due to a high frequency of certain haplotypes in clearwater habitat (Fig 6C). In conclusion, the selective sweep on a single haplotype carrying a red-shifted *SWS2* cone opsin coding sequence and linked noncoding variation in *SWS2* and *LWS* may be associated with successful repeated colonization of blackwater habitat across the Haida Gwaii adaptive radiation.

**Table 2:** General linear model results for genotype–environment association tests.

	T400			Depth			Area		
Gene	β	t_4,50_	*p*	β	t_4,50_	*p*	β	t_4,50_	*p*
*SWS1*	**0.16**	**4.09**	**<0.01**	−0.02	−0.44	0.66	0.15	0.43	0.67
*RH2*	−0.02	−0.41	0.68	0.05	0.81	0.42	−1.26	−2.75	0.01
*SWS2*	**-0.10**	−**3.36**	**<0.01**	0.07	1.72	0.09	0.16	0.60	0.55
*LWS*	−**0.06**	−**3.39**	**<0.01**	0.04	1.97	0.06	0.00	0.02	0.99
	Clear- versus Blackwater			Depth			Area		
Gene	β	t_4,50_	*p*	β	t_4,50_	*p*	β	t_4,50_	*p*
*SWS1*	2.44	2.46	0.02	0.03	0.52	0.60	−0.08	−0.22	0.83
*RH2*	−0.81	−0.68	0.50	0.06	0.91	0.37	−1.26	−2.82	0.01
*SWS2*	−**2.91**	−**4.37**	**<0.01**	0.06	1.79	0.08	0.23	0.92	0.36
*LWS*	−**1.33**	−**3.50**	**<0.01**	0.03	1.70	0.10	0.05	0.38	0.71

The upper half of the table shows test results with light spectrum as a continuous variable, the lower half shows test results with light spectrum as a categorical variable: clearwater versus blackwater (T400 ≤ 74%). Bold numbers indicate significant tests after Bonferroni-correction for multiple testing.

The selection experiment supports a role of the sweep haplotype and associated coding variation in *SWS2* during adaptation to different light regimes: The blackwater-associated *SWS2* haplotype with the red-shifted *SWS2* allele occurs at high frequency in the blackwater population, Mayer Lake, where a selective sweep signature is persistent (Figs 2–5). In contrast, the frequency of the alternate haplotype has rapidly increased after 13 generations in the clearwater habitat, with blue-shifting *SWS2* key site substitutions rising from 13% to 40% frequency, with significant population differentiation, and with positive Tajima’s D centered on *SWS2* (Figs 4 and 5). Shifts in allele frequency at *SWS2* key site substitutions and linked regulatory sites ranks them among the top 5% and 1%, respectively, for allele-frequency change across the genome in the selection experiment (Fig 4), making the blue-shifted *SWS2* haplotype a genome-wide outlier and thus a likely target of reversed selection due to habitat shift. Under a pure selection model, an allele-frequency shift of 27% over approximately 13 generations would correspond to an evolutionary change of 1.12 haldanes and to a selection coefficient of 0.28 (see materials and methods). The experimental transfer of a blackwater population to a clearwater habitat thus lead to evolution in the expected direction, given the habitat association across the adaptive radiation: a change from red- to blue-shifted *SWS2* allele after the transfer into clearwater habitat.

### Evolutionary origin of the red-shifted *SWS2* haplotype in stickleback

**Fig 8:**
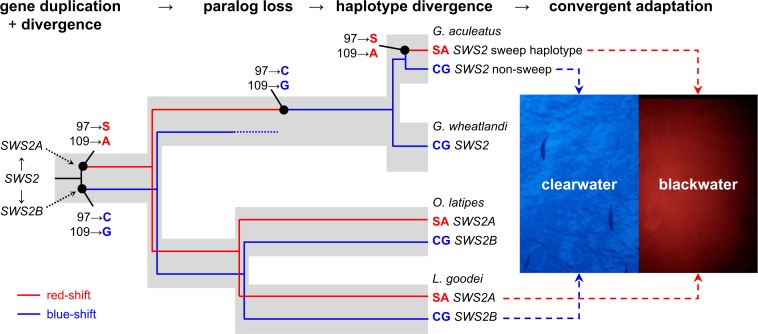
Convergent evolution of the blue-sensitive *SWS2* opsin at the molecular, functional, and ecological level. The duplication of *SWS2* in the ancestor of most spiny-rayed fish 198 million years ago was followed by a red-shift in *SWS2A* and a blue-shift in *SWS2B* [22, 47], paralogs that are divergently expressed among bluefin killifish (*L*. *goodei*) living in blackwater and clearwater habitats [24]. Two key amino acid polymorphisms of the ancient paralogs causing shifts in their absorption spectra have reevolved within threespine stickleback and are now divergently selected between blackwater and clearwater habitats in Haida Gwaii—convergent evolution at the molecular, functional, and ecological level. Clearwater spectra (left photo) are blue-shifted with increasing depth, typical of marine habitats and oligotrophic clearwater lakes on Haida Gwaii [18]. Tannin-stained blackwater (right photo) absorbs almost all up- and sidewelling light, making it a nearly nocturnal habitat, except for red-shifted downwelling light visible in a small cone above the observer. Unedited photos taken with a GoPro Hero 4 (GoPro Inc.) pointed towards the zenith at approximately 20 m depth in clearwater (Palau) and at 4 m depth in blackwater (Drizzle Lake).

The blue light–sensitive *SWS2* gene has undergone two duplication and divergence cycles [47], of which the first, in the ancestor of spiny-rayed fish, has led to a blue-shifted *SWS2B* paralog and a red-shifted *SWS2A* paralog [22, 26, 53, 56–58]. The single *SWS2* gene copy of threespine stickleback is derived from an *SWS2A* paralog, while the other paralogs have been lost in the stickleback lineage [22, 47]. Strikingly, the two *SWS2* key site amino acid polymorphisms found in our study are also key sites that have led to red- and blue-shifts in the *SWS2A* and *SWS2B* paralogs, respectively (Fig 6B, [47, 53]): at these two key sites, the sweep haplotype in threespine stickleback shows the same amino acids as the ancestral red-shifted *SWS2A* paralog, and the alternate haplotype shows the same amino acids as the blue-shifted *SWS2B* paralog lost in the stickleback lineage (Fig 8). Also, at three of the remaining five *SWS2* substitutions segregating among threespine stickleback (sites 33, 150 and 169), the amino acids on the sweep haplotype are the same as in *SWS2A* paralogs of medaka (*Oryzias latipes*) and bluefin killifish (*Lucania goodei*), while some amino acids on the nonsweep haplotype are identical to their *SWS2B* paralogs.

We tested whether the similarity of the red- and blue-shifted stickleback *SWS2* haplotypes with each of the ancestral paralogs was due to shared ancestry, gene conversion, or convergent evolution. First, we reconstructed the evolutionary origin of the two threespine stickleback *SWS2* haplotypes to ask whether both threespine stickleback haplotypes are derived from an *SWS2A* ancestor. For this, we embedded the two most common *SWS2* threespine stickleback haplotypes (*n*_sweep_ = 74, *n*_non-sweep_ = 23 of 116 haplotypes from all 58 sequenced individuals, Fig 7) into a phylogeny with an orthologous *SWS2* sequence from blackspotted stickleback (*Gasterosteus wheatlandi*) and both *SWS2A* and *SWS2B* paralogs from shorthorn sculpin (*Myoxocephalus scorpius*), medaka, and bluefin killifish. We chose these taxa because their *SWS2* paralogs have not been excessively affected by gene conversion [47]. The phylogeny confirmed that both threespine stickleback *SWS2* haplotypes and blackspotted stickleback *SWS2* were derived from an *SWS2A* paralog, in line with findings of earlier studies on the origin of the stickleback *SWS2* [22, 47], ruling out shared ancestry as an explanation for the similarity between *SWS2* paralogs and stickleback haplotypes (Fig 6B).

Second, we asked whether gene conversion between *SWS2A* and *SWS2B* paralogs in the lineage leading to threespine stickleback may have contributed to the similarity between *SWS2* paralogs and the threespine stickleback *SWS2* haplotypes. For this, we computed divergence at synonymous sites (dS) between the threespine stickleback haplotypes and both paralogs of the shorthorn sculpin in sliding windows across the gene (Fig 6D). The divergence distribution shows that the *SWS2B* paralog is more divergent from both stickleback haplotypes than the *SWS2A* paralog almost throughout the whole gene, inconsistent with ancestral gene conversion. A single region of reduced synonymous divergence in the last third of the protein suggests ancient gene conversion but does not overlap with the amino acid polymorphisms among Haida Gwaii stickleback. This gene conversion signal has been ascribed to the shorthorn sculpin lineage and not the threespine stickleback lineage in a larger phylogenetic analysis [47]. Ancestral gene conversion thus cannot explain key site similarity between segregating stickleback haplotypes and divergent ancestral paralogs. Instead, new, recurrent mutations in threespine stickleback must have led to convergent amino acid changes with the ancestral paralogs.

We tested whether the *SWS2* sweep haplotype in threespine stickleback has accumulated amino acid–changing mutations at an extraordinarily rapid rate (“positive selection”). Branch-specific dN/dS estimates in the *SWS2* phylogeny show an elevated dN/dS ratio of 1.64 on the branch leading to the selective sweep haplotype (Fig 6B), indicative of accelerated amino acid substitution and positive selection. A branch-site model test for positive selection on protein sequence, however, could not distinguish between positive selection on this branch and a null model without positive selection (ΔLRT = 0.65, *p* = 0.13). As the terminal threespine stickleback branches contain only a few substitutions (N * dN = 5.2 and S * dS = 1.0 on the sweep haplotype branch and N * dN = 1.9 and S * dS = 1.0 on the nonsweep haplotype branch), the low divergence of the two haplotypes has likely limited our power to distinguish the two models using the branch-site test, which is most powerful for divergent sequences from interspecific comparisons [59, 60].

## Discussion

Our study reveals that threespine stickleback have adapted wavelength sensitivity through selection at the *SWS2* locus. A single red-shifted *SWS2* allele has been favored across the adaptive radiation and blackwater lakes, most of which were colonized in replicate from marine ancestors and are almost exclusively inhabited by individuals with this red-shifted *SWS2* allele. The evolution of a red-shifted *SWS2* opsin thus likely facilitated the colonization of blackwater lakes and subsequent establishment in this extreme habitat. Tannin-stained blackwater is characterized by a red-shifted light spectrum with reduced transmission of short wavelengths [18]. A blue-sensitive opsin spectrally tuned to a longer wavelength will thus increase an individuals’ ability to detect any residual short wavelength light in blackwater. Such an adaptation mechanism is supported by genomic signatures of selection, by functional effect predictions and by genotype-environment associations across the adaptive radiation and in the selection experiment. Also, previously observed phenotypic differences [18] support this mechanism: cones expressing *SWS2* in blackwater stickleback from some of these and other populations had an absorption spectrum red-shifted by ~10 nm [18], a stronger shift than is explainable by alternate chromophore use. The combined results from our study and Flamarique et al. [18] thus show that threespine stickleback adapted visual perception to blackwater habitats by spectral tuning of *SWS2* key sites, causing a higher sensitivity to the remaining short wavelength light, and increased expression of *LWS* in double cones, maximizing the sensitivity to background light.

Remarkably, the molecular mechanism underlying this recent adaptation in threespine stickleback recapitulates the duplication and divergence of *SWS2* around 198 million years ago in the spiny-rayed fish ancestor [22, 53]. Amino acid replacements at *SWS2* key sites are identical and thus convergent between the threespine stickleback *SWS2* alleles and the *SWS2A* and *SWS2B* paralogs, respectively [18, 47, 53]. Such convergent spectral tuning at key sites of cone opsins has been found previously but exclusively at larger evolutionary timescales, such as between damselfish species [40], between butterflies and vertebrates [41], butterflies and bees [42], humans and poeciliid fish [31], or across the animal kingdom [43]. Convergent spectral tuning between such vastly different time scales—on one side, a microevolutionary, intraspecific level and on the other side, a 198 million-y-old duplication-divergence process—has not yet been shown to our knowledge.

Not only the mechanism of spectral tuning at *SWS2* is convergent, but also the environmental context: two other fish species inhabiting both tannin-stained blackwater and clearwater habitats, bluefin killifish and black bream *Acanthopagrus butcheri*, show expression divergence between the red-shifted *SWS2A* and blue-shifted *SWS2B* paralogs [24, 26, 37, 61]. Bluefin killifish populations occur either in blackwater or clearwater habitats [24, 37], and black bream live in clearwater as juveniles and migrate to blackwater habitats where they spend their adult life [26, 61]. In both species, the red-shifted *SWS2A* cone abundance is higher in blackwater habitats and the blue-shifted *SWS2B* cone abundance is higher in clearwater habitats, which is due to reduced *SWS2B* expression and an increased *SWS2A* expression relative to *SWS2B*, respectively [24, 61]. While the two key amino acids at sites 97 and 109 each are convergent between bluefin killifish paralogs and stickleback alleles living in either blackwater or clearwater (Fig 8, [37, 47]), black bream has substituted these with other amino acids but still shows the same function for the two paralogs (red- and blue-shift) [26] and thus ecological, phenotypic, and functional convergence.

Adaptation to blackwater environment via *SWS2* spectral tuning and therein improved visual capacities can have a multitude of consequences for survival and reproduction. The light environment in blackwater lakes is limited to downwelling, red-shifted light and thus visual detection of predators and prey is much reduced, leading to short action and reaction distances. Any improved detection of prey or predators, for example, via increased sensitivity to color contrast at residual short wavelengths, would be favored by natural selection. Bluefin killifish from blackwater environments indeed showed increased color contrast attention towards blue objects in blackwater [62]. Increased color contrast attention might also be favored by sexual selection: the “blue morph” in bluefin killifish is more abundant in blackwater, and blue males are preferred by individuals raised in stained water [63, 64]. Similarly, stickleback inhabiting blackwater systems have lost red nuptial throat color and instead show black throats, contrasting with the background and with blue eyes, and these two traits are under sexual selection by choosy females [17, 19]. Spectral tuning of blue-sensitive *SWS2* may thus be under both natural and sexual selection in threespine stickleback and other blackwater-adapted fish species.

Our selection experiment confirmed that the segregating *SWS2* alleles are favored by selection in different light environments: after only 13 generations in a clearwater habitat, the red-shifted *SWS2* allele associated with the blackwater sweep haplotype decreased in frequency while the alternate blue-shifted allele swept to high frequency, indicating a reverse, ongoing sweep in clearwater habitats. The direction of change is in line with both functional predictions and genotype–environment association across the adaptive radiation. The evolutionary change of 1.12 haldanes estimated from this allele-frequency shift is much larger than the change in feeding morphology or predator defense morphology traits, which show a mean change of 0.22 haldanes over 12 generations [48]. This could arise from inherent differences between genotype- and phenotype-based estimates, such as the increased variation due to a complex genetic basis and environmental effects in phenotypic estimates [65]. Change in allele frequency at *SWS2* by 27% is comparable to the strongest relative changes in trait means: gill raker length was reduced by 43%, lateral plate three height by 18%, and lateral plate two frequency and dorsal spine length by 16% [48]. Selection on color vision thus led to similarly rapid or slightly faster evolutionary change compared to other, feeding and predator defense–related traits; and genetic and phenotypic change was in the direction predicted from independently evolved populations across the adaptive radiation, recapitulating the same habitat contrast.

Repeated use of the same red-shifted *SWS2* haplotype during replicated adaptation to blackwater lakes in Haida Gwaii suggest that these adaptive mutations have been present as standing genetic variation in the marine population prior to colonization. Indeed, the marine individual in our dataset is heterozygous for all *SWS2* amino acid polymorphisms, confirming the presence of both red-shifted sweep and blue-shifted nonsweep haplotypes in a marine population (Fig 2). Also, freshwater populations outside Haida Gwaii, such as one individual from mainland British Columbia in our study (Table 1, Figs 5 and 7) and the reference genome, a female freshwater stickleback from Alaska, carry the same red-shifted *SWS2* haplotype, while another mainland individual carries the blue-shifted *SWS2* haplotype. Maintenance of two spectrally tuned opsin alleles as standing genetic variation might be a “microevolutionary rescue” solution to the loss of multiple functionally divergent *SWS2* paralogs in the lineage leading to threespine stickleback, which could explain convergent evolution with the ancestral *SWS2* paralogs. To maintain such divergent *SWS2* alleles, more complex selection scenarios than selection in blackwater might be necessary, including disruptive or fluctuating selection or selection for a red-shifted allele in other freshwater habitats than blackwater lakes. Visual spectra in freshwater habitats rapidly change with depth, dissolved organic particles, and other biophysical properties, making more complex scenarios likely. Further study of cone opsin variation in additional freshwater and marine populations, taking ecological knowledge of visual environments into account, may provide better insight into the maintenance of variation and repeatability of adaptation to light spectra.

By combining population genomic data, functional genomic analysis and a selection experiment, we have uncovered the genetic mechanisms underlying repeated adaptation of color vision to divergent visual environments in threespine stickleback. This mechanism of adaptation is convergent at the molecular, functional, and ecological level with other fish species that have used 198 million-y-old paralogs to adapt to similar blackwater environments. Convergent evolution at the same gene happened thus at vastly different timescales, involving two mechanisms: repeated de novo mutation, leading to convergent amino acid changes, and the reuse of standing genetic variation for repeated adaptation in an adaptive radiation. Our study thus supports the emerging view that mechanisms underlying adaptive evolution are often highly repeatable and likely predictable [4] and that evolutionary tinkering with the same, constrained toolset can lead to convergent adaptation, both within species and between distantly related groups.

## Materials and methods

### Ethics statement

Stickleback collection followed guidelines for scientific fish collection in British Columbia, Canada, under Ministry of Environment permits SM09-51584 and SM10-62059. Collections in Naikoon Provincial Park and Drizzle Lake Ecological Reserve were carried out under park use permits: 103171, 103172, 104795, and 104796.

### Sampling, sequencing, and variant calling

Among the more than 100 stickleback populations previously studied from the Haida Gwaii archipelago [16], a subset of 25 populations was chosen to comprise the full range of biophysical attributes of the freshwater habitats on Haida Gwaii, including water spectra, lake area, bathymetry, and predation regime. Stickleback from these 25 freshwater sites, two freshwater sites in coastal British Columbia [66], and one marine site were collected between 1993 and 2012, using minnow traps or recovery from salmon stomachs (marine sample, Table 1, for coordinates, see [16, 66]; coordinates BKW2: 53.375089°N, −130.177378°W). We selected one to four individuals per population for whole-genome resequencing. From the selection experiment [48], we chose 12 fish from the source population Mayer Lake and 11 from the population introduced into Roadside Pond (equivalent to “Mayer Pond” in [48]), the latter sampled in 2012, 19 y or approximately 13 generations after the release of 100 Mayer Lake fish, assuming a generation time of 1.5 y being intermediate between Mayer Lake (2 y generation time) and Roadside Pond (1 y generation time). In total, 58 individuals, 56 females and two males, were resequenced to 6x depth using paired-end Illumina reads as described in [6] at the Broad Institute.

We aligned reads to the Broad S1 reference [6] using BWA v0.5.9 [67] with parameters -q 5 -l 32 -k 2 -o 1 and recalibrated base qualities using the GATK v1.4 tools CountCovariates and TableRecalibration [68], with read group, quality score, cycle, and dinucleotide covariates in the recalibration model. This resulted in 2,992,040,331 aligned and recalibrated reads. Variants were called using GATK’s UnifiedGenotyper for each chromosome separately and all 58 individuals combined, with default parameters for SNP and indel calling, respectively. We removed variants with quality normalized by depth ≥ 2, read position rank sum test value ≥ −20, and allele-specific strand bias ≤ 200, using GATK’s VariantFiltration from the dataset. We recalibrated variants using the GATK’s VariantRecalibrator and ApplyRecalibration with a VQSR-LOD cutoff of 98.5%. Filtered and recalibrated variants were lifted over to an improved ordering of scaffolds in the reference stickleback genome [51] using Picard v2.2.1 (http://broadinstitute.github.io/picard). Also, we realigned base quality recalibrated reads to this improved reference using samtools v1.3 [69] and BWA v0.7.12 with the same alignment parameters as above, in order to enable read-backed phasing and read-based genotype likelihood–based analyses (see below), resulting in 2,935,821,595 aligned reads, which have been deposited on the NCBI short read archive under accession SRP100209.

We obtained a set of high-quality SNPs by removing all variants failing variant recalibration, variants with quality < 45 and with a mean depth > 9.51 (= average mean depth plus 1.5 times the interquartile range of the mean depth distribution), variants with less than four reads of each allele, variants with more than two alleles, and indels, using bcftools v1.3.1 [69]. This dataset was partitioned by chromosome, and males (individuals in populations Banks 70, Laurel) were removed from the sex chromosome XIX partition. SNPs were further split into an “adaptive radiation” and “selection experiment” SNPs partition. The “adaptive radiation” SNP partition contained one randomly picked individual from each of the 28 populations except the transplant population Roadside Pond (Table 1) in order to perform further analyses with equal sample size for all natural populations. The “selection experiment” SNP partition contained all 12 and 11 individuals from Mayer Lake and Roadside Pond, respectively. In both adaptive radiation and selection experiment SNP datasets, genotypes with less than four reads and sites with more than 50% missing genotypes were removed using vcftools v0.1.15 [70], resulting in 15.3% and 16.2% missing genotypes, respectively. Both the adaptive radiation and selection experiment SNP datasets were phased and missing genotypes imputed with the read-backed phasing algorithm implemented in SHAPEIT v2.r790 [71]. Phase-informative reads covered 9.3% of all heterozygote genotypes and 32.7% of all graph segments.

### Population genomic analyses

We scanned the genomic regions containing the four cone opsins for signatures of selective sweeps by using variation across the whole genome to identify outlier regions. We computed two haplotype-based statistics, integrated haplotype score iHS and H12 [49, 50], for the phased adaptive radiation SNPs. These statistics have been developed to detect signatures of incomplete hard and soft selective sweeps, based on extended haplotype homozygosity around an allele under selection compared to its alternate allele (iHS, [49]) or based on the haplotype frequency spectrum expected under a selective sweep (H12, [50]). Applied to the adaptive radiation dataset, these statistics will capture selective sweeps shared by multiple members of the adaptive radiation. iHS for each SNP in the genome was computed in selscan v1.1.0b [72] with default parameters and standardized in 5% allele frequency bins. In addition, we calculated the percentage of absolute iHS values > 2 in nonoverlapping 10 kb windows with more than 10 iHS estimates [49]. H12 was computed in bins spanning 81 SNPs using scripts published alongside the definition of H12 [50]. We used an outlier approach to identify significant iHS and H12 regions. We identified the top 0.1% genome-wide outliers for SNP-iHS, window-iHS, and H12 in recombination rate bins (<0.5, 0.5–2, 2–3.5, 3.5–5, >5 cM/Mb) because of the sensitivity of these statistics to variation in recombination rate. Local recombination rates were estimated from the “FTC x LITC”-cross recombination map published in [51] with a cubic splines smoothing approach described in [73]. For a significant outlier region indicating a selective sweep centered on opsins *SWS2* and *LWS*, we used the top H12 estimates to identify the haplotype under selection or “sweep haplotype.” We visualized haplotype structure around a selective sweep in both adaptive radiation and selection experiment datasets using the extended haplotype homozygosity (EHH) statistic [74] calculated in selscan with default parameters.

We further traced the evolution of the selective sweep region around *SWS2* and *LWS* in the selection experiment. For this, we computed population differentiation (F_ST_) between Mayer Lake and Roadside Pond as well as nucleotide diversity (π) and Tajima’s D (T_D_) in each population across the genome and linkage disequilibrium (r^2^) in the selective sweep region for the unphased selection experiment SNPs. We first estimated the folded two-dimensional site-frequency spectrum (2D-SFS) from genotype likelihoods at all sites, from aligned reads with mapping-quality ≥ 17 and bases with quality ≥ 17 using angsd v0.911 [75, 76]. Using this 2D-SFS, we computed π and T_D_ in 10 kb nonoverlapping as well as 10 kb wide, 2 kb step sliding windows across the genome for each population using angsd [76]. Then we estimated population allele frequencies with angsd and used them with the 2D-SFS to compute F_ST_ in 10 kb nonoverlapping as well as 10 kb wide, 2 kb step sliding windows across the genome using realSFS from the angsd software package [76, 77]. As for iHS and H12, we identified the top 0.1% outliers among nonoverlapping windows, based on the genome-wide distribution of π and T_D_ in recombination rate bins. We computed linkage disequilibrium (r^2^) across the selective sweep region for SNPs with minor allele frequency (MAF) ≥ 5% and maximum 20% missing data in both populations using vcftools.

We identified synonymous- and nonsynonymous variation in the coding sequence of the four cone opsins *SWS1*, *SWS2*, *RH2*, and *LWS* in both the adaptive radiation and selection experiment SNPs. For the adaptive radiation SNPs, we tested whether coding variation at cone opsins was associated with the sweep haplotype identified above by using both alleles at each nonsynonymous SNP and chi-square tests with Bonferroni-corrected *p*-values. We also estimated the mean ratio of pairwise sequence divergence at synonymous and nonsynonymous sites (mean pairwise dN/dS) for all pairs of haplotypes in the adaptive radiation dataset using PAML v4.8 [78] and following the approach by Yang and Nielsen [79]. This statistic measures the relative frequency of segregating amino acid polymorphism to silent mutations [80]. We assessed whether any of the four cone opsins showed an unusually high frequency of amino acid changes by computing the distribution of mean pairwise dN/dS for all functional amino acid–coding genes on assembled chromosomes in our dataset (*n* = 17,846 genes).

### Genotype–environment association

We tested whether genetic variation at the four cone opsins was associated with variation in light spectrum, using three environmental proxies of light spectrum, percent light transmission at 400 nm (T400), lake depth in meters, and log-transformed lake area in square meters. We assigned SNPs to up- and downstream regulatory regions, introns, exons, and 3′/5′-untranslated regions of each cone opsin gene using SnpEff v4.2 [81] and combined all SNP alleles per gene into a single multidimensional scaling (MDS) coordinate in R v3.3.1 [82]. We used the MDS coordinate as a response variable in a general linear model with three predictor variables: T400, lake depth, and log-transformed lake area. To test more specifically for an association with blackwater environment, we repeated the general linear model analysis with a categorical light transmission variable “clearwater” for lakes with T400 > 74% and “blackwater” with T400 ≤ 74%, following [16]. Significance of effects was determined after Bonferroni-adjustment for multiple testing. We qualitatively assessed which SNPs are most strongly correlated with blackwater habitat from single-SNP chi-square tests for each gene-associated SNP.

For the selection experiment populations Mayer Lake and Roadside Pond, we estimated allele frequencies based on genotype likelihoods with angsd v0.911 [75] using raw-aligned reads of mapping quality > 17 for sites with quality > 17 and the GATK genotype likelihood model [68]. We computed allele-frequency changes for all variable sites in the genome and the empirical quantiles for absolute allele-frequency changes at SNPs surrounding *SWS2* in Mayer Lake–based MAF bins of width 0.05. We also computed evolutionary change in haldanes at *SWS2* key sites following equation 1 in [65], with raw allele frequency mean, standard deviations, and a generation time of 12.7 as input. Furthermore, we estimated the expected selection coefficient under a pure selection model, following equation 3.2 in [83], assuming incomplete dominance h = 0.5 and using a per generation allele-frequency change by dividing the observed allele-frequency change by 12.7 generations.

### Evolutionary history of *SWS2*

We reconstructed the evolutionary history of the cone opsin associated with a selective sweep, *SWS2*, using a Bayesian phylogenetic approach implemented in MrBayes v3.2.6 [84] and the same evolutionary model and run parameters as in [47]. For phylogenetic reconstruction, we used the two most common threespine stickleback *SWS2* haplotypes, one associated and the other not associated with the sweep haplotype; an *SWS2* sequence of blackspotted stickleback ([85], SRA-accession DRR013347); and both *SWS2A* and *SWS2B* paralogs from shorthorn sculpin ([47], genbank accession KM978046.1), medaka ([56], genbank accessions AB223056.1, AB223057.1), and bluefin killifish ([53], genbank accessions AY296737.2, AY296736.1). To get the blackspotted stickleback *SWS2* sequence, we aligned whole-genome sequence data with a subset of the threespine stickleback reference sequence spanning the *SWS2* coding sequence and 1 kb sequence up- and downstream of the start and stop codon, respectively, using bowtie2 v2.2.3 [86] with parameters -N 1 and -L 20, called genotypes using the GATK v3.5 tool UnifiedGenotyper [68] with default parameters and genotype likelihood model “BOTH,” converted the variants to FASTA format using the GATK-tool FastaAlternateReferenceMaker, and reintroduced missing sites using bedtools v2.25.0 [87]. We then aligned threespine stickleback and blackspotted stickleback *SWS2* with the *SWS2A* and *SWS2B* paralogs of the other species manually in BioEdit v7.2.5 [88] and clipped the alignment to codons present in all sequences. We created the same alignment of codons for all 116 phased threespine stickleback haplotypes in our dataset to compute a neighbor-joining network using standard parameters in PopART v1.7 (http://popart.otago.ac.nz).

We tested whether selection on the threespine stickleback *SWS2* haplotype lead to a rapid accumulation of amino acid–changing mutations compared to synonymous mutations (“positive selection”) on this haplotype. We used the phylogeny from above, estimated branch-specific dN, dS, and dN/dS and performed a branch-site test for positive selection with a subset of the phylogeny containing only stickleback *SWS2* and other species’ *SWS2A* paralogs [60, 89], using PAML v4.8 [78] and the threespine stickleback’s sweep-associated haplotype as the foreground branch. Following [47], we also tested for gene conversion between *SWS2A* and *SWS2B* paralogs in the threespine stickleback ancestor by calculating dS between threespine stickleback *SWS2* haplotypes and shorthorn sculpin *SWS2A* and *SWS2B* paralogs in 30 bp sliding windows with 1 bp step size in DnaSP v.5.10.01 [90]. After excluding gene conversion, we assessed whether amino acid substitutions at opsin key sites in the stickleback *SWS2* haplotypes paralleled the divergence of ancestral *SWS2A* and *SWS2B* paralogs. We also added a partial coding sequence of black bream *SWS2A* and *SWS2B* paralogs ([26], genbank accessions DQ354580.1, DQ354581.1) to assess potential molecular and functional convergence with two other species inhabiting both clear- and blackwater (black bream, bluefin killifish).

## Supporting information

S1 Fig Linkage disequilibrium around *SWS2* and *LWS* in the selection experiment. Both axes give positions along chromosome XVII and boxes on top and right of the figure indicate the position of genes.
(TIFF)

S1 Data Figs 1–6 data. (XLSX)

## Abbreviations

dN/dS: ratio of non-synonymous to synonymous substitutions
EHH: extended haplotype homozygosity
MAF: minor allele frequency
