# Supplementary figures and images for: Convergent evolution of SWS2 opsin facilitates adaptive radiation of threespine stickleback into different light environments
Source: PLoS Biol. 2017 Apr 11;15(4):e2001627. doi: 10.1371/journal.pbio.2001627 (PMC5388470; doi:10.1371/journal.pbio.2001627)

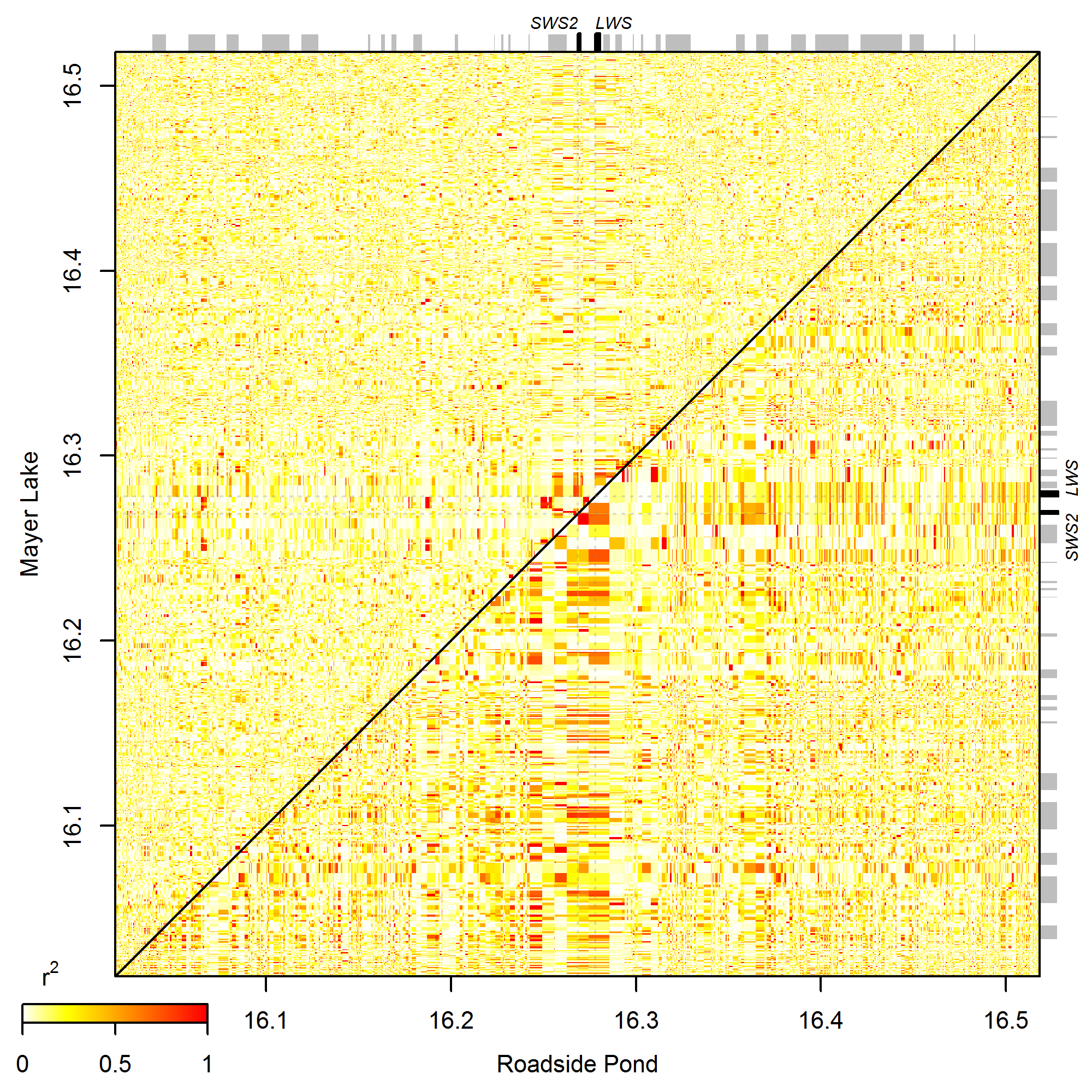

Supplement: S1 Fig — Both axes give positions along chromosome XVII and boxes on top and right of the figure indicate the position of genes. (TIFF) [file pbio.2001627.s001.tiff]
